# Tunicate mitogenomics and phylogenetics: peculiarities of the *Herdmania momus *mitochondrial genome and support for the new chordate phylogeny

**DOI:** 10.1186/1471-2164-10-534

**Published:** 2009-11-17

**Authors:** Tiratha Raj Singh, Georgia Tsagkogeorga, Frédéric Delsuc, Samuel Blanquart, Noa Shenkar, Yossi Loya, Emmanuel JP Douzery, Dorothée Huchon

**Affiliations:** 1 Department of Zoology, George S Wise Faculty of Life Sciences, Tel-Aviv University, Tel Aviv 69978, Israel; 2 Institut des Sciences de l'Evolution (UMR 5554), CNRS-Université Montpellier II, Place Eugène Bataillon, 34095 Montpellier Cedex 05, France; 3 Laboratoire d'Informatique, de Robotique et de Microélectronique de Montpellier (UMR 5506), CNRS-Université Montpellier II, 161 rue Ada, 34392 Montpellier Cedex 05, France; 4 Department of Biology, University of Washington, Seattle WA 98195, USA

## Abstract

**Background:**

Tunicates represent a key metazoan group as the sister-group of vertebrates within chordates. The six complete mitochondrial genomes available so far for tunicates have revealed distinctive features. Extensive gene rearrangements and particularly high evolutionary rates have been evidenced with regard to other chordates. This peculiar evolutionary dynamics has hampered the reconstruction of tunicate phylogenetic relationships within chordates based on mitogenomic data.

**Results:**

In order to further understand the atypical evolutionary dynamics of the mitochondrial genome of tunicates, we determined the complete sequence of the solitary ascidian *Herdmania momus*. This genome from a stolidobranch ascidian presents the typical tunicate gene content with 13 protein-coding genes, 2 rRNAs and 24 tRNAs which are all encoded on the same strand. However, it also presents a novel gene arrangement, highlighting the extreme plasticity of gene order observed in tunicate mitochondrial genomes. Probabilistic phylogenetic inferences were conducted on the concatenation of the 13 mitochondrial protein-coding genes from representatives of major metazoan phyla. We show that whereas standard homogeneous amino acid models support an artefactual sister position of tunicates relative to all other bilaterians, the CAT and CAT+BP site- and time-heterogeneous mixture models place tunicates as the sister-group of vertebrates within monophyletic chordates. Moreover, the reference phylogeny indicates that tunicate mitochondrial genomes have experienced a drastic acceleration in their evolutionary rate that equally affects protein-coding and ribosomal-RNA genes.

**Conclusion:**

This is the first mitogenomic study supporting the new chordate phylogeny revealed by recent phylogenomic analyses. It illustrates the beneficial effects of an increased taxon sampling coupled with the use of more realistic amino acid substitution models for the reconstruction of animal phylogeny.

## Background

Mitochondrial genomes (mtDNAs) of bilaterian animals are short, circular DNA molecules of 14-16 kb in length, typically characterized by the absence of introns and the presence of only short intergenic regions, with the exception of the control region (CR), a non-coding region assumed to contain the elements for the regulation of replication and transcription of the genome [1,2]. To date, about 1,700 complete metazoan mitochondrial genomes have been sequenced and used in comparative mitogenomics and phylogenetic studies on different taxonomic scales [3-9].

The mitochondrial gene content is highly conserved across the different bilaterian phyla, with typically 37 genes [1,2,10]. Among them, 13 genes encode for proteins [ATP synthase subunits 6 and 8 (*atp6 *and *atp8*), cytochrome oxidase subunits (*cox1*, *cox2*, *cox3*), apocytochrome b (*cytb*), and dehydrogenase subunits (*nd1*, *nd2*, *nd3*, *nd4*, *nd5*, *nd6 *and *nd4L*)]. The remaining genes encode two ribosomal subunits (*srRNA *and *lrRNA*) and usually 22 tRNAs. However, cases of duplication and loss of tRNAs have been reported within bilaterians [11,12]. Tunicate mitochondrial genomes illustrate such exceptions and typically encode 24 tRNAs, apart from two *Phallusia *species which lack the tRNA-Asp [13], and *Halocynthia roretzi *which encode two tRNA-phe [14]. The two additional tRNAs present in tunicate mitochondrial genomes when compared to vertebrates are (i) the tRNA-Gly (for AGR codons), which is necessary for the translation due to the derived tunicate mitochondrial genetic code [15], and (ii) the tRNA-Met (for AUA codon), whose presence might reduce the conflict between translation initiation -- which requires a tRNA-Met (for AUG) -- and translation elongation that involves AUG codons [16].

The mitochondrial gene order is highly conserved within Deuterostomia [10,17], and particularly in chordate genomes. Conversely, mitochondrial gene arrangement shows an important plasticity in some animal phyla, (*e.g*. molluscs and nematodes [1,2]), and especially in tunicates [2,13,18-22]. Tunicates, or Urochordates, are marine deuterostomes characterized by markedly diversified developmental and life history traits, and traditionally encompass three major classes: Ascidiacea (sea squirts), Thaliacea (salps) and Appendicularia (larvaceans). Ascidiacea, commonly referred to as ascidians, is the most speciose and widespread group. Several ascidian species have been identified as invasive species, such as *Styela clava *and *Pyura praeputialis *[23-26], and have a strong ecological impact on the invaded marine ecosystems. Some species are also widely used as model organisms in evo-devo studies like *Ciona intestinalis *and *Botryllus schlosseri *[27-31]. According to the traditional classification, the class Ascidiacea is subdivided into three major orders: Phlebobranchia, Aplousobranchia, and Stolidobranchia. In contrast with this taxonomic view, 18S rRNA-based phylogenies have shown that ascidians are in fact paraphyletic [32-34]. According to the 18S rRNA phylogenetic framework, Aplousobranchia, Phlebobranchia, and Thaliacea are closely related, whereas Stolidobranchia forms a distinct and monophyletic group, which might be close to Appendicularia, although the position of the latter is still debated [32,33].

To date, complete mitochondrial genomes of tunicates are mainly available for a single representative of Thaliacea (*Doliolum nationalis*) and five ascidians [13,18-22], including four phlebobranchians (*Ciona intestinalis *type A and B, *C. savignyi*, *Phallusia fumigata*, *P. mammillata*) and one stolidobranchian (*Halocynthia roretzi*). The available mitochondrial data suggest that several unique features characterize mitogenomic evolution in tunicates relative to other chordate phyla. Two main peculiarities can be distinguished. The first refers to the highly variable gene order observed within the group, which implies that extensive gene rearrangements have occurred even at low taxonomic levels [13,18,19]. However, since most available tunicate complete mtDNA sequences belong to phylogenetically-related species (except *H. roretzi*) according to the 18S rRNA reference [32-34], it is not possible to evaluate whether mitochondrial gene rearrangements characterize the whole order or only the Aplousobranchia + Phlebobranchia + Thaliacea clade. The second specificity is that of an accelerated evolutionary rate of tunicates, as revealed by the long branches of the group in mitogenomic topologies [35-37] and the associated composition bias [37]. However, whether this accelerated substitution rate is restricted to protein coding genes as in snakes [38] or is a more general feature of the whole mtDNA of Tunicates, has yet to be investigated.

These two peculiar evolutionary features of tunicate mitochondrial genome evolution have hampered their reliable phylogenetic placement within metazoans. Analyses of mitochondrial protein-coding genes have almost always systematically placed tunicates outside Bilateria [20,35,36,39]. This is in sharp contrast with recent nuclear-based phylogenomic studies that identified tunicates as the closest living relatives of vertebrates within chordates [40-43]. Only two recent mitogenomic study have found marginal support for chordate monophyly. Bourlat et al. [37] grouped cephalochordates with vertebrates according to the traditional Euchordata hypothesis using a concatenation of the 13 protein coding genes under a site- and time-heterogeneous mixture model in Bayesian phylogenetic reconstructions. Alternatively, Zhong et al. [39] recovered the new chordate phylogeny under the maximum likelihood framework when removing the fastest evolving vertebrates species and when considering only the four most conserved mitochondrial proteins.

Here, we sequenced the complete mitochondrial genome of the solitary ascidian *Herdmania momus *(Ascidiacea: Stolidobranchia: Pyuridae), an Indo-Pacific species that was introduced into the Mediterranean Sea via the Suez Canal [44]. We describe the structural and compositional features of *H. momus *mtDNA, discuss its evolutionary dynamics with respect to other tunicate and chordate mitochondrial genomes, and provide an updated metazoan phylogeny based on probabilistic analyses of the 13 mitochondrial proteins using site- and time-heterogeneous mixture models of amino acid substitutions.

## Results and Discussion

### General features of *H. momus *mtDNA

**Figure 1:**
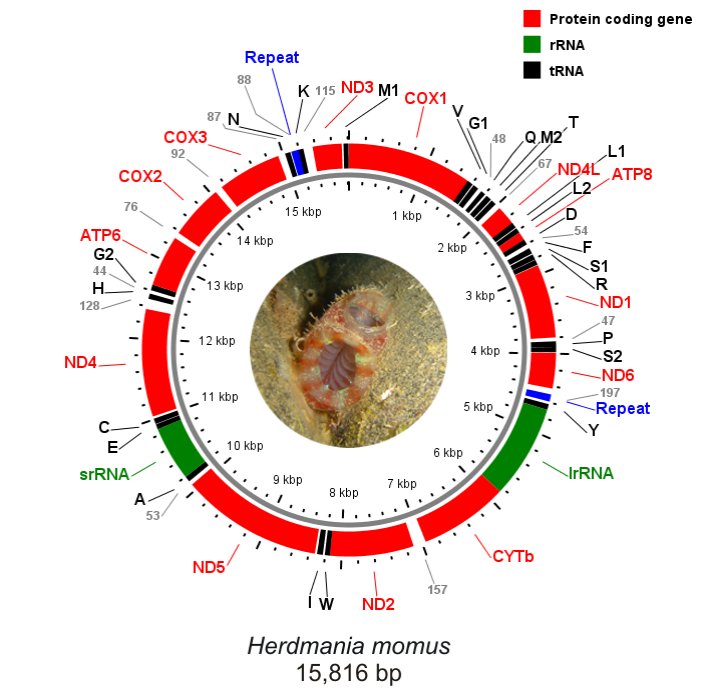
***Herdmania momus *mitochondrial genome map**. Protein coding genes, rRNAs, and tRNAs are shown in red, green, and black, respectively. Gray numbers indicate the length of non coding regions longer than 40 bp, the location of the repeated sequences is indicated in blue.

**Figure 2:**
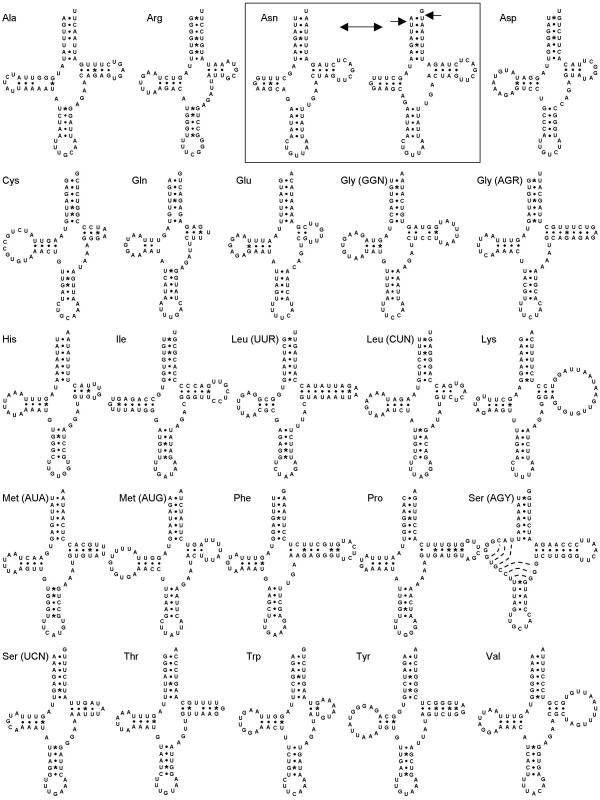
**Putative cloverleaf structures for the 24 tRNA genes of the *Herdmania momus *mitochondrial genome**. Canonical and G-T base pairs are indicated by dots and asterisk respectively. The two boxed tRNA-Asn correspond to the tRNA-scan predicted structure (left) and a less stable alternative structure (right).

The mitochondrial genome of *H. momus *(Figure 1) accounts for 15,816 base pairs (bp) in length which falls within the typical range of other tunicate genomes; the smallest genome being 14,579 bp long in *P. fumigata *and the largest one 16,351 bp in *D. nationalis*. It presents the typical tunicate gene content with all 13 protein-coding genes of the mitochondrial respiratory apparatus, including *atp8*, which appears so divergent in Tunicates that it had been difficult to annotate in the initial assemblies of *C. savignyi *and *H. roretzi *[18,21,22]. It also encodes for the two mitochondrial ribosomal genes, *srRNA *and *lrRNA*, and a total of 24 tRNAs (Figure 2), among which are two distinct tRNAs for the Gly (AGR and GGN), Leu (UUR and CUN), and Ser (AGY and UCN) codons (Figure 2), which is consistent with the modified mitochondrial genetic code of tunicates. Finally, *H. momus *mtDNA contains an additional tRNA-Met (AUA), similar to all other tunicate genomes sequenced so far [13,18-22].

In contrast to other deuterostome mtDNA genomes, all genes are encoded on the same DNA strand and thus mitochondrial gene transcription shows only one orientation in *H. momus*, as in all other tunicate genomes sequenced to date. In chordates, although the majority of genes is co-orientated on one major DNA strand (H-strand), a minimum set of tRNAs and the *nd6 *gene are encoded from the minor one (L-strand) [2,17,45]. The genome architecture of *H. momus *thus provides additional evidence in support of the hypothesis that gene arrangement in only one strand is a shared derived feature of tunicate mtDNAs [2].

With regard to genome organization, *H. momus *mtDNA seems substantially less compact than other available tunicate mitochondrial genomes. Adjacent genes overlap in four cases: *cox1 *- tRNA-Val (9 bp), *nd4L *- tRNA-Leu (15 bp), *atp8 *- tRNA-Asp (1 bp) and *nd2 *- tRNA-Trp (2 bp). The total gene overlap thus appears slightly lower with respect to the average ~35 bp gene overlap of the other tunicate genomes [13]. Interestingly, there is no protein-protein gene overlap. Furthermore, *H. momus *shows a high percentage of non-coding (NC) sequence (ca. 10%).

Concerning base composition, the mitochondrial genome of *H. momus *is AT rich with a value of 63.5%. The overall base frequencies as estimated in the coding strand follow the decreasing order T, G, A and C. T is by far the most abundant nucleotide with 41.3% in the genome. Most tunicate mtDNAs present similar compositional AT-rich profiles varying from 61.1% in *D. nationalis *to 78.6% in *C. intestinalis*. The only exception concerns the mitochondrial genomes of species from the genus *Phallusia*, which present more balanced AT and GC compositions with 52.8% and 53.2% AT in *P. mammillata *and *P. fumigata*, respectively [13].

### Protein-coding genes

The predicted protein-coding genes in the mtDNA of *H. momus *present lengths that are overall similar to their orthologues in the other tunicate genomes. Nevertheless, *H. momus *was not predicted to carry any deletion at the C-terminal ends of *nd4 *and *nd5 *genes similar to those reported in *H. roretzi *[21].

The base composition of protein-coding genes in *H*. *momus *was measured as being A+T rich, as is the profile of its entire genome. Consequently, the codon usage follows the same pattern, with C-rich codons showing lower frequencies than those that are T- or G- rich. Extreme cases are constituted by the CGC codon for Arginine, which is never used, and by the UUU codon for Phenylalanine and UUG codon for Leucine, which are the most frequently used. The CGC codon is also never used in the two *Ciona *genomes, whereas it is present in other tunicate genomes [18,22].

The preferred start and stop codons in *H. momus *mitochondrial genes are GTG (6 genes) and TAG (7 genes), respectively. In the other Pyuridae (*H. roretzi*), GTG is the preferred start codon too, but TAA is the most frequent stop codon (8 genes). Like in other tunicate genomes, incomplete T-starting stop codons are predicted at the end of the three genes coding for *nd1*, *nd4 *and *nd4L*. The complete TAA stop codon is probably created by polyadenylation.

### rRNA genes

Because of high sequence divergence of tunicate rRNA sequences, the boundaries of all tunicate mitochondrial rRNA sequenced so far have been inferred from the flanking genes. Likewise, the *srRNA *gene in *H. momus *is estimated at most as 673 bp long, thus being slightly shorter than its acidian orthologues which range from 687 to 738 bp, and about 30 bp longer than the *srRNA *of *D. nationalis *mtDNA. On the genome map, it is found located between the tRNA-Ala and tRNA-Glu. The *lrRNA*, on the other hand, appears to be at most 1,159 bp long, a length similar to that of other tunicates, and is located downstream of tRNA-Tyr and upstream of *cytb *(Figure 1).

Both lengths and locations of the two rRNA genes in the *H. momus *mitochondrial genome provide additional evidence for two unique features of mtDNA evolution in tunicates. The first is that tunicates present the shortest mitochondrial rRNA genes among chordates, and more generally among deuterostomes, with lengths varying at most from 641 to 738 bp for the small ribosomal subunit and from 1,059 to 1,279 bp for the large ribosomal subunit [13]. The corresponding lengths for the *srRNA *and *lrRNA *genes in the other chordate lineages are estimated as higher than 844 bp and 1,367 bp, respectively. The second feature shared by all tunicates concerns the location of the two genes in the mitochondrial genome. In contrast to all other chordates, where the two rRNA coding genes are usually adjacent, *srRNA *and *lrRNA *are found rearranged and separated in all available tunicate mtDNAs [2]. The distance between the two genes in *H. momus *genome is about 5 kb. Distances of the same order of magnitude (5-7 kb) were also identified in the mtDNAs of Cnidaria, some Protostomia, and some Echinodermata [2].

### tRNA genes

The predicted cloverleaf structures for the 24 tRNA genes of the *H. momus *mitochondrial genome are presented in Figure 2. As in *H. roretzi*, only two tRNAs are characterized by an unusual structure: tRNA-Ser (AGY) and tRNA-Asn. tRNA-Ser (AGY) appears to possess an 8 bp-long anticodon stem structure. Similar long stems (7-9 bp) have been reported in *Ciona *ssp.*, D. nationalis*, and *H. roretzi *tRNA-Ser (AGY) [18,20-22]. However, this structure is absent in *Phallusia *tRNA-Ser (AGY), which appears to have retained the classical cloverleaf conformation [13]. Concerning the tRNA-Asn gene, two alternative cloverleaf structures have been inferred, as also previously reported for *H. roretzi *[21]. The structure predicted by tRNA-scan (Figure 2) is characterized by a two-nucleotide spacer between the DHU and the anti-codon stem as well as a mismatch at the start of the T stem. The alternative conventional cloverleaf structure (Figure 2) appears less stable, in forming a mismatch at the start of the D arm. Similar two-nucleotide spacers have also been found in the tRNA-Asn structures of *Phallusia *ssp., *H. roretzi*, and *D. nationalis*, but not in *Ciona *ssp [13,18,20-22].

### Non-coding regions

Table 1 summarizes some attributes of the non-coding (NC) regions in *H. momus *and the other available tunicate mitochondrial genomes. In total, 28 NC regions have been identified in *H. momus *mtDNA, with a total size of 1,457 bp (9.21% of the total genome). When compared to other ascidians, the proportion of NC regions in *H. momus *appears higher, with previous estimates varying from 1.94 to 5.89%, in the mtDNAs of *P. mammillata *and *P. fumigata*, respectively. The proportion of NC sequences in the closely-related *H. roretzi *is three-fold lower (3.09%) than that of *H. momus*, yet the total number of NC fragments is almost identical in both species, and the two genomes differ by ca. 1 kb in length. Among tunicates, only *Doliolium nationalis *has more non-coding regions with 13.1% of the total genome length (Table 1).

**Table 1:** Statistics on non-coding (NC) sequences in tunicate mitochondrial genomes.

**Species**	**% NC**	**Length** **(bp)**	**Longest NC region**	
			**bp**	**location**
	
***Ciona intestinalis***	2.9	429	85	*nd1 *- *lr*RNA
***Ciona savignyi***	2.9	428	194	*nd1 *- tRNA Pro
***Phallusia fumigata***	5.89	915	134	*cox1 *- tRNA Gly (GGN)
***Phallusia mammillata***	1.94	283	65	tRNA Cys - *nd4L*
***Doliolium nationalis***	14.71	2405	968	*cox3 - cox2*
***Halocynthia roretzi***	3.09	456	112	*nd4 *- tRNA Val
***Herdmania momus***	9.21	1457	197	*nd6 *- tRNA Tyr

* including 1 bp-long spacers.

Considering length distributions, 19 of the 28 NC regions present lengths over 20 bp, with the longest accounting for 197 bp and being located between the *nd6 *and tRNA-Tyr genes (Figure 1). Three additional NC regions measuring more than 100 bp have been identified in *H. momus *mtDNA, involving the disjunction of the following gene pairs: *cytb *- *nd2*, *nd4 *- tRNA-His, and tRNA-Lys - *nd3*. The remaining nine NC sequences are shorter intergenic spacers, mostly 3-10 bp long, distributed homogenously through the genome.

The presence of palindrome sequences was checked by sequence similarity searches and has been detected within 12 NC regions, and in particular within the three longest NC regions of the *H. momus *mitochondrial genome. However, no stem-loop structure similar to that of the control region of vertebrate mt-genomes has been identified in *H. momus*. Further studies are necessary to establish whether these regions are involved in the control of replication and transcription.

Sequence similarities searches have finally revealed the occurrence of a duplication event in the NC regions of the *H. momus *genome, similar to those previously determined in *Ciona intestinalis *and *Phallusia fumigata *mtDNAs [13]. Here, a fragment of 89 bp was found duplicated in two different locations of the genome: the first, situated in the non-coding region downstream of the *nd6 *gene and upstream of the gene encoding for the tRNA-Tyr (NC = 197 bp); while the second involved the non-coding region downstream of the tRNA-Asn (NC = 88 bp) and 13 bp of the 5' region of the tRNA-Lys. The estimated distance between the repeated motifs is about 5 kb (Figure 1).

### Gene order

**Figure 3:**
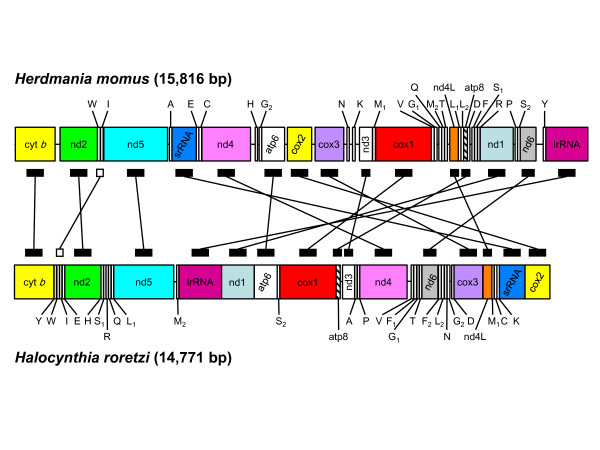
**Comparison of mitochondrial gene order in Stolidobranchia**. The gene orders in the mitochondrial genomes of the two stolidobranchs *Halocynthia roretzi *and the newly-obtained *Herdmania momus *are compared. To illustrate the extent of the gene order rearrangements, the same protein-coding genes are connected by black rectangles between the two stolidobranchian mitochondrial genomes. The only syntenic region between the two related genomes represented by a single gene pair, including tRNA-Trp - tRNA-Ile, is indicated by two connected white rectangles.

The mtDNA of *H. momus *shows a novel gene arrangement with respect to other tunicates, and is radically different from that of its close relative *Halocynthia roretzi *(Figure 3). Surprisingly, only one block containing two consecutive genes is conserved between the two species: the pair tRNA-Trp - tRNA-Ile. When considering only the relative arrangement of protein-coding genes, an additional block appears to be shared by the two members of Stolidobranchia, consisting of the three genes *cytb *- *nd2 *- *nd5*. In *H. roretzi*, *cytb *and *nd2 *are separated by four tRNA-coding genes, whereas in *H. momus *the two genes are separated by a long non-coding region of 157 bp. Likewise, the number of tRNA genes separating *nd2 *and *nd5 *as well as their identities differ in each genome (two and five tRNAs separate these two genes in *H. momus *and *H. roretzi *respectively). Conversely, the pair *cox2*- *cytb*, previously considered as unique and shared by all other tunicates is absent in *H. momus*, as well as the pair tRNA-Arg - tRNA-Gln which is conserved in most tunicate genomes[13,18-22].

Thus, the mitochondrial genome organization in *H. momus *when compared to that of *H. roretzi *suggests that extensive gene rearrangements occur within Stolidobranchia, similar to those observed in Phlebobranchia [13]. Future work should determine whether co-generic species in Stolidobranchia also present high variations in gene order, as reported for the genera *Ciona *and *Phallusia *within Phlebobranchia [2].

### Phylogenetic analyses

The mitochondrial genome has been repeatedly used in molecular phylogenetics of animals, principally due to several convenient features, such as its relatively small size, its cellular abundance, and its mostly uniparental mode of inheritance facilitating orthology assignment [5]. However, the phylogenetic resolving power of mtDNAs is often reduced by pervasive lineage-specific variations of base composition and substitution rate [46,47]. Bilaterian evolutionary relationships obtained from previous phylogenetic reconstructions based on mitochondrial genes [35,36] are in relatively good agreement with the currently accepted view of Protostomia phylogeny [48]. However, the situation is more complex concerning Deuterostomia, mainly because of the recurrent placement of tunicates as sister-group of either the remaining Bilateria [35,36] or the other chordates [37]. Such placement outside chordates is in direct contradiction to results from recent large-scale phylogenomic studies, which strongly support the clade Olfactores, *i.e*., the grouping of tunicates with craniates (jawed vertebrates + cyclostomes) to the exclusion of cephalochordates [40-43]. The sister position of tunicates relative to the remaining Bilateria is generally interpreted as a long-branch attraction artifact caused by the peculiarities of tunicate mitogenomic evolution in terms of both lineage-specific evolutionary rate and amino-acid composition [36,37,39].

**Figure 4:**
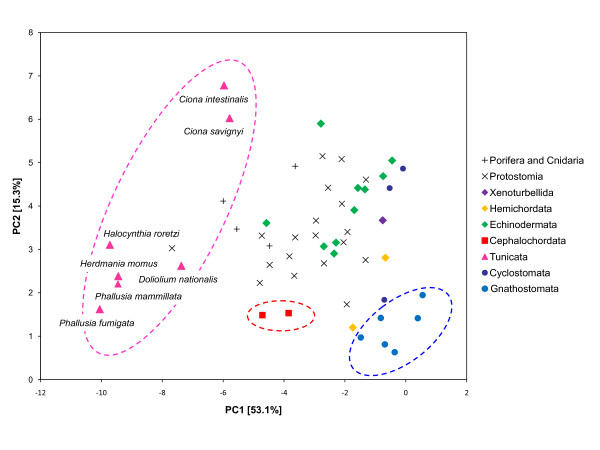
**Analysis of amino acid composition heterogeneity of mitochondrial proteins among metazoans**. Principal Component Analysis (PCA) of the amino acid composition of the 13 mitochondrial proteins from the 54-taxon metazoan dataset. Individuals are plotted in the first two principal components of the PCA which explain 53.1% and 15.3% of the total compositional variance, respectively. Points corresponding to Cephalochordata (red), Tunicata (purple) and Gnathostomata (blue) are circled.

In our mitogenomic dataset, the potential occurrence of compositional biases was explored through a Principal Component Analysis (PCA) of amino acid composition of the 54 taxa (Figure 4). This statistical analysis shows that tunicates have a markedly heterogeneous amino acid composition that is clearly distinct from most other sampled taxa. Moreover, tunicates appear extremely divergent from the other chordate representatives. Tunicates and jawed-vertebrates are located at the extreme opposite sides of the graph, with cephalochordates in an intermediate position. There is thus a strong compositional heterogeneity in our amino-acid dataset that might cause phylogenetic artefacts if it is not specifically accounted for in models of sequence evolution.

The maximum likelihood tree obtained on the 13 protein-coding genes concatenation using the site- and time-homogeneous mtREV+Γ_4 _model of amino-acid sequence evolution places tunicates as the sister group of the remaining Bilateria, as previously reported [35,36,39]. However, the bootstrap support for such a position is low (BP = 61). This result is presumably the outcome of two types of systematic biases: compositional heterogeneity of the tunicate lineage compared to other chordate and animal phyla (see Figure 4) combined with accelerated evolutionary rates [37,39]. ML phylogenetic analyses were also conducted under an empirical site-heterogeneous CAT mixture model [49] which has been shown to alleviate long-branch attraction artefacts in the context of animal phylogenomics [50]. The ML tree inferred under this model shows a dramatic topological shift in placing tunicates as the sister-group of craniates within monophyletic chordates and deuterostomes, in agreement with phylogenomic studies [35,41]. However, this phylogenetic placement is not statistically supported (BP < 50).

In order to further explore the effect of model misspecification on mitogenomics tree inference, Bayesian analyses have been conducted using a range of amino-acid sequence evolution models (Table 2). There is a clear distinction between the results obtained using site-homogeneous models (mtREV+Γ_4 _and GTR+Γ_4_) on the one side, and variants of the CAT+Γ_4 _site-heterogeneous model [51] and the site- and time-heterogeneous CAT+BP+Γ_4 _model [52], on the other side. Site-homogeneous models moderately support a sister-group relationship between tunicates and *Xenoturbella *(PP ≥ 0.83), whereas the different CAT mixture models support the grouping of tunicates with craniates (*i.e*., monophyly of Olfactores) with the highest Bayesian posterior probabilities obtained using the most complex and better-fitting models (PP ≥ 0.94). These results exemplify the impact of the model of sequence evolution on mitogenomic inference of phylogeny by showing that the new chordate phylogeny [35,40,41] can be corroborated by using a model that accounts for both compositional and evolutionary rate heterogeneities.

**Table 2:** Bayesian posterior probabilities (PP) for alternative positions of tunicates using different models of amino-acid replacements.

Model	Tunicata + Craniata	Tunicata + *Xenoturbella*	Tunicata sister to the remaining Bilateria	Tunicata + Euchordata*	Tunicata + Ambulacraria	Tunicata sister to the remaining Deuterostomia
**mtREV+Γ**_**4**_ (12,000 trees)	0.14	**0.85**	0	<0.01	0	<0.01
**GTR+Γ**_**4**_ (24,000 trees)	0	**0.83**	0.16	0	0	<0.01
**CAT** **+Poisson+Γ**_**4**_ (240,000 trees)	**0.71**	0.08	<0.01	0.11	0.02	0.02
**CAT** **+mtREV+Γ**_**4**_ (12,000 trees)	**0.96**	0	0	<0.01	0	0
**CAT** **+GTR+Γ**_**4**_ (12,000 trees)	**0.95**	0	0	<0.01	<0.01	0
**CAT** **+BP+Γ**_**4**_ (34,800 trees)	**0.94**	0.03	0	0.02	<0.01	<0.01

* Euchordata = Cephalochordata + Craniata

PP were obtained from 3 independent MCMC runs for all models except CAT+BP+Γ_4 _for which 5 MCMC were run. The number of trees used to compute PP is indicated.

**Figure 5:**
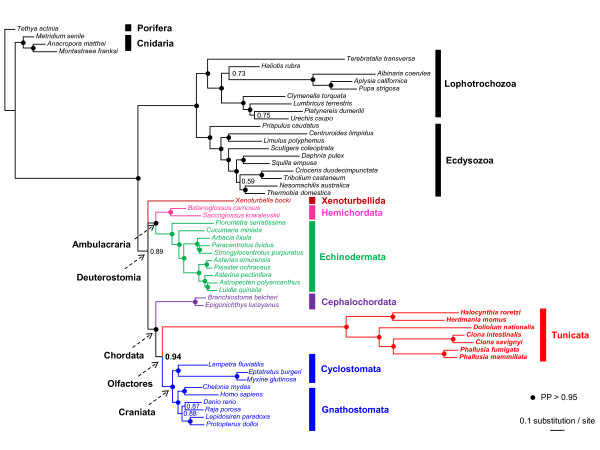
**Phylogeny of Metazoa inferred from the concatenation of the 13 mitochondrial proteins**. Bayesian consensus tree of 5 individual MCMC obtained using the CAT+BP+Γ_4 _mixture model on the concatenation of the 13 proteins (54 taxa and 2,136 amino-acid sites). Values at nodes indicate Bayesian posterior probabilities (PP). Circles indicate strongly supported nodes with PP ≥ 0.95. The scale bar represents the estimated number of substitutions per site.

The Bayesian consensus tree obtained under the site- and time-heterogeneous CAT+BP+Γ_4 _model is detailed in Figure 5. The overall phylogenetic picture is consistent with the common tripartite structure of Bilateria phylogeny [53], with Protostomia and Deuterostomia, and Protostomia being further divided into Lophotrochozoa and Ecdysozoa. Strong statistical support is obtained for the monophyly of Protostomia (PP = 1), and for both of its two major lineages Lophotrochozoa and Ecdysozoa (PP ≥ 0.99). The monophyly of Deuterostomia is less strongly supported however (PP = 0.89), in agreement with recent mitogenomic [37] and phylogenomic results [41,54]. Within Deuterostomia, Echinodermata and Hemichordata were both retrieved as firmly monophyletic (PP = 1) and are strongly grouped into Ambulacraria, as suggested by early mitogenomic studies [55] and later confirmed by phylogenomics [35,41,43]. However, the position of *Xenoturbella *within Deuterostomia remains unresolved in our analysis (PP < 0.5) as also found in the latest mitogenomic analysis [37], whereas phylogenomics support its sister-group relationship with Ambulacraria into a clade named Xenambulacraria [35,41]. Strong statistical support is obtained for the respective monophyly of the three chordate groups: Cephalochordata (PP = 1), Tunicata (PP = 1), and Craniata (PP = 0.99). The monophyly of Chordata is strongly supported (PP = 0.96) for the first time in a mitogenomic study, as is the monophyly of Olfactores (PP = 0.94). These results are fully congruent with the growing body of evidence coming from phylogenomic studies that revealed the unexpected sister-group relationship between tunicates and craniates [35,39,40,42,43].

Although removal of fast-evolving gene and taxa has been found to recover the new chordate phylogeny, such an approach is not successful to solve relationships within Tunicata [39]. Our mitogenomic analysis provided strong evidence for intra-tunicate relationships, with the overall phylogenetic scheme being well resolved despite the high lineage-specific evolutionary rate (Figure 5). The inferred relationships for the group were notably in concordance with nuclear-based phylogenies and morphological data. More precisely, *Herdmania *was unambiguously retrieved in a sister-group relationship with *Halocynthia *(PP = 1), in agreement with the systematic classification that places these two ascidian genera within the stolidobranch family Pyuridae [33]. Firm evidence was similarly obtained for the respective grouping of the congeneric species of *Ciona *and *Phallusia *(PP = 1), and high posterior probability is retrieved for a sister-group relationship of the two genera into Phlebobranchia (PP = 1). Finally, the thaliacean *Doliolum *is found to branch within ascidians with solid statistical support (PP = 0.99), favoring the hypothesis of Ascidiacea paraphyly [20] as also supported by 18S rRNA phylogenies [32,34,56].

### Compared evolutionary rates of rRNA- and protein-coding genes

**Figure 6:**
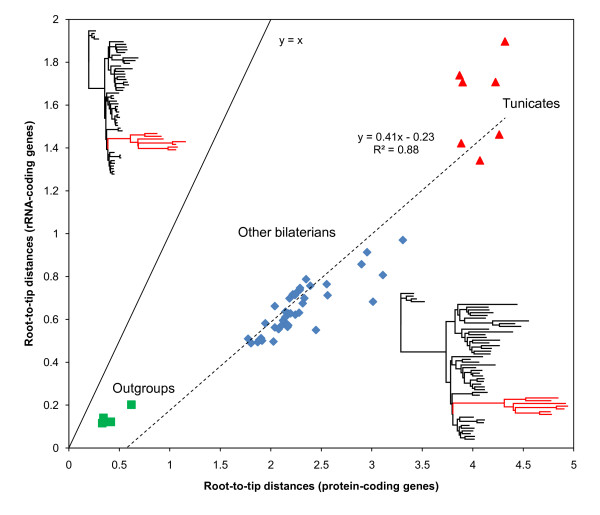
**Comparison of lineage-specific evolutionary rates**. The graph is a plot of the 54 root-to-tip distances calculated from branch lengths estimated under the GTR+Γ_8 _model on the reference topology (see Figure 5) for the concatenations of rRNA-coding (y-axis) and protein-coding (x-axis) mitochondrial genes. Symbols corresponding to non-bilaterian outgroups (green squares), tunicates (red triangles), and all other bilaterians (blue diamonds) are indicated. The phylograms showing the branch lengths inferred from each dataset are presented with the highly evolving tunicates figured in red. The linear regression dotted line is figured with its associated equation and correlation coefficient. The y = x line is also indicated to underline the difference in average evolutionary rate between the rRNA and protein mitochondrial partitions.

A detailed analysis of lineage-specific evolutionary rate was performed by inferring branch lengths on the Bayesian consensus reference phylogeny (see Figure 5). The comparison of branch lengths obtained on the protein- and rRNA-coding mitochondrial partitions, first demonstrates that tunicates are clearly the fastest evolving species of the metazoan dataset on both partitions (Figure 6). It also shows that the rRNA-coding partition evolves on average about three times more slowly than the protein-coding gene partition (TBL ratio = 34.79/11.76 = 2.96). However, in contrast to what has been observed in snake mitochondrial genomes [38,57], there is very good correlation between root-to-tip distances inferred from the two partitions (R^2 ^= 0.88). These results reveal that tunicate mitochondrial genomes have experienced a drastic acceleration of evolutionary rate that affects both protein-coding genes and ribosomal-RNA genes. This latter observation seems therefore to exclude the occurrence of adaptive evolution episodes in protein-coding genes, recently reported for snake and agamid lizard mitochondrial genomes [58], as the ultimate cause behind the elevated substitution rates in tunicate mitochondrial genomes. Nevertheless, the lineage-specific rate acceleration of tunicates probably explains the difficulties previously encountered in reconstructing their phylogenetic position based on mitogenomic data.

## Conclusion

The complete mitochondrial genome of the ascidian *H. momus *shares several features with the other tunicate mtDNAs so far sequenced: (i) all genes are encoded on the same strand; (ii) two additional tRNAs, when compared with vertebrates, are present (one for Gly, one for Met); (iii) the gene order is highly rearranged, and differs from all other known tunicates, with the noticeable synteny disruption of the *cox2*--*cytb *gene block; and (iv) the evolutionary rate is elevated and involves both protein- and rRNA-coding genes. From the phylogenetic viewpoint, the analysis of the concatenated mitochondrial proteins recovers the tunicates + craniates clade within monophyletic chordates, provided that site- and time-heterogeneous mixture models of amino acid replacements are used.

## Methods

### Sampling and DNA extraction

The *H. momus *(Savigny, 1816) individual used for our study was collected in June 2006 on the Eilat-Ashkelon pipeline pier in Eilat, Red Sea 29°31' N 34°55' E at a depth of 12 m [44]. Genomic DNA was isolated from gonads by placing ~0.5 cm^3 ^of tissue in 1 ml of lysis buffer (100 mM Tric-HCl pH = 7.8; 100 mM EDTA; 0.5% SDS; 0.2 mg/ml proteinase K) overnight at 55°C. The digestion was followed by Phenol:Chloroform:Isoamyl alcohol extraction (25:24:1) and isopropanol precipitation [59].

### DNA sequencing and gene annotation

Primers were designed to match conserved regions of chordate genomes in the *cox1, cox2, cox3*, and *cytb *genes (Table S1 in Additional File 1). PCR amplifications were performed using the ExTaq polymerase (TaKaRa) in 25 μL reaction mixture according to manufacturer's instructions and were conducted in two steps. A first amplification was performed with external primers followed by a re-amplification of the initial PCR product using different sets of internal primers. Based on the partial sequences obtained for the aforementioned genes, longer specific primers were subsequently designed. This allowed us to determine the *cox3-cox1 *region and to redesign (long) primers in the *nd3 *gene located in the same region.

The complete mitochondrial genome was finally amplified in two overlapping fragments using the Long and Accurate (LA) Taq polymerase (TaKaRa) in 25 μL reaction mixture according to manufacturer's instructions. The first fragment covered the *cox3-cytb *region (~7,700 bp) and was obtained by PCR amplification using the external primers HMO_Cox3_Long_F1 5'-ACTGTTGTTTTACTTAGTTCGGGAGTTACTGTGAC-3' and HMO_Cob_Long_R1 5'-ACATATAAGCGACCCCCAACAAAAAGAAAC-3' followed by a re-amplification with the primers HMO_Cox3_Long_F2 5'-AGTTTTGGTGGAGGGCTTATGGGATTAGTTTGGAC-3' and HMO_Cob_Long_R2 5'-GACACCAGAATAGGGCCAAAAATAAATCTTT-3'. The second fragment encompassed the *cytb-nd3 *region (~8,900 bp) and was similarly acquired by a first PCR amplification using the external primers HMO_Cob_Long_F1 5'-AGGGGTGCTACTTTAACGCGGTTTTACAC-3' and HMO_Cox1_Long_R2 5'-ACATTATACAACTGCCCATCTCCAATTACCTG-3' and a subsequent re-amplification using the primers HMO_Cob_Long_F2 5'-AGTGGTTTTGTCTTTGGTGCATTTGGTTTTC-3' and NADH3_R 5'-CTGAATAGAATAACCCTCGCTATCACT-3'. The two fragments overlapped over ~150 bp at 5' end of the *cytb *and over ~780 bp at the *cox3-nd3 *region. The sequencing of the long PCR products was performed by the company MACROGEN (Seoul, Korea) using shotgun sequencing (i.e., shearing of the PCR fragment, bacterial library construction, and sequencing of the clones). Contigs were assembled using both ARACHNE [60] and Chromaspro (Technilysium Pty. Ltd.) programs. Regions of low coverage in the assembly (i.e., fewer than three clones) were confirmed by primer-walking sequencing (~4 kb of the genome was re-sequenced) with the use of specific primers and genomic DNA as template. The complete mtDNA sequence of *H. momus *was deposited under the EMBL accession number FN296153.

Protein coding genes were identified using the ORF-Finder tool of NCBI [61] and the DOGMA server [62]. Following Iannelli et al. [13], boundaries of protein coding gene were inferred in such a way that overlap between genes was minimized and similarity between tunicate proteins was maximized. ATG and non-standard initiation codons [63] were considered as reliable start codons.

Both the tRNAscan-SE 1.23 program and the DOGMA server were used to identify and infer the secondary structures of tRNA genes [64]. tRNAs that were not identified by the above tools were sought by folding all putative non-coding regions using the Mfold server [65]. All predicted cloverleaf secondary structures were manually checked and compared with other known ascidian tRNAs. The positions of the small (*srRNA*) and large (*lrRNA*) ribosomal RNA genes were identified by the DOGMA server and confirmed based on sequence similarity searches to orthologous genes in other ascidians. Finally, the boundaries of the rRNA genes were inferred from the flanking genes.

### Dataset assembly

The phylogenetic dataset was built upon the taxon sampling used by Bourlat *et al*. [35]. We expanded the tunicate sampling to seven by including the newly-sequenced *H. momus *and two recently published genomes from the genus *Phallusia *[13]. The snake representative (*Boa constrictor*) was excluded because snake mitochondrial genomes have been shown to be the subject of adaptive evolution that can severely bias phylogenetic inference [58]. The final dataset therefore comprises 54 taxa including 31 deuterostomes, 19 protostomes, and four non-bilaterian outgroups (*cf*. Additional file 1). The nucleotide sequences of all mitochondrial genes were retrieved from the Organellar Genome Retrieval (OGRe) database [66]. For the 13 protein coding genes, sequences were translated, and aligned at the amino-acid level using MAFFT with default parameters [67]. Ambiguously aligned sites were identified and removed from all individual genes separately, using the program Gblocks [68] with the following parameters: minimum number of sequences for a conserved position = 18; minimum number of sequences for a flanking position = 29; maximum number of contiguous nonconserved positions = 8; minimum length of a block = 2; allowed gap positions = with half. The concatenation of the 13 proteins yielded a phylogenetic dataset including 54 taxa and 2136 unambiguously aligned amino-acid sites, of which 1697 were variable (Additional file 2).

### Phylogenetic analyses

Phylogenetic analyses were conducted using Maximum Likelihood (ML) and Bayesian Inference (BI) reconstruction approaches on the concatenated amino acid dataset. Maximum Likelihood (ML) analyses were performed using the program PHYML 3 [69] under the models mtREV+Γ_4 _and CAT+Γ_4_, with a 4-category Gamma (Γ_4_) distribution of the among-site amino acid replacement rate heterogeneity [70], and with the number of CAT categories set to 20 (C20) as recommended by Le et al. [49]. The heuristic ML searches were conducted by performing Subtree Pruning and Regrafting (SPR) moves on a Neighbor-Joining (NJ) starting tree. Statistical support was estimated by Bootstrap resampling with 100 pseudo-replicates generated by the program SeqBoot of the PHYLIP package [71]. In all replicates, ML analyses were performed using PHYML through the same heuristic search strategy. Nodal bootstrap supports (BS) were obtained from the 50% majority rule consensus of the 100 reconstructed trees using the program TREEFINDER [72].

Bayesian inference was conducted using the program PhyloBayes 3.1 [73] under both homogeneous amino acid models (mtREV+Γ_4 _and GTR+Γ_4_) and variants of the site-heterogeneous CAT+Γ_4 _mixture model (CAT+Poisson+Γ_4_, CAT+mtREV+Γ_4_, and CAT+GTR+Γ_4_) [51]. The program nhPhyloBayes [74] was used to performed Bayesian analysis under a site- and time-heterogeneous model which combines the break-point approach (BP) in order to model variations of amino acid replacement rates along branches and the CAT mixture model in order to account for site-wise variations of these rates. In this CAT+BP+Γ_4 _analysis, the number of categories of the mixture component was fixed at 60 (C60), and the biochemical profiles were those inferred by Le et al. [49], rather than being estimated.

In Bayesian analyses under mtREV+Γ_4_, GTR+Γ_4_, and under the different variants of CAT+Γ_4 _(CAT+Poisson+Γ_4_, CAT+mtREV+Γ_4_, and CAT+GTR+Γ_4_), three independent Markov Chain Monte Carlo (MCMC) were run in parallel, whereas five MCMC were run for analyses under CAT+BP+Γ_4_. Each MCMC was launched from a random initialization and for a large number of cycles with parameters and trees saved every cycle. Priors were set to values as described in Blanquart and Lartillot [52] concerning the CAT+BP model, and in Lartillot et al. [73] concerning all other applied models. Convergence of MCMC was checked by monitoring the marginal likelihood through cycles. Bayesian Posterior Probabilities (PP) were obtained from the trees sampled during the stationary phase of the different MCMC.

### Branch length analysis

Two concatenated nucleotide datasets were constructed to compare evolutionary rates of protein-coding and rRNA-coding mitochondrial genes. In both cases, the different genes were individually aligned using MAFFT, and ambiguously aligned sites were removed using Gblocks before building each concatenation. This led to a 13 protein-coding gene dataset containing 8,130 nucleotide sites and a rRNA dataset including 806 sites (Additional files 3 and 4). Using the rooted topology obtained from the analysis of the amino-acid sequences under the CAT+BP+Γ_4 _mixture model as a reference, ML branch lengths were optimized under the GTR+Γ_8 _model using PAUP* 4.0b10 [75] on both the protein-coding and rRNA-coding nucleotide concatenations. For each dataset, the root-to-tip distance was calculated for each of the 54 taxa by summing the branch lengths on the path going from the root of the tree to each of its terminals. The two sets of root-to-tip distances were then compared graphically to evaluate the lineage-specific rate correlation between the two mitochondrial partitions.

## Authors' contributions

FD, EJPD and DH conceived and initiated the study. YL and NS collected and identified the biological specimens. TRS carried out DNA extraction, PCR amplifications, and sequencing. TRS and DH annotated the mitochondrial genome. GT, SB and FD performed the phylogenetic analyses. FD, EJPD, YL, and DH supervised the work in their respective labs. GT, FD, EJPD and DH wrote the manuscript. All other authors assisted in revising the manuscript. All authors read and approved the final manuscript.

## Supplementary Material

Additional file 1 **Species sampling, taxonomy and sequence accession numbers**. The table indicates the taxonomy and the species sampling used in the present study with associated sequence Accession Numbers for their complete mitochondrial genomes. Click here for file

Additional file 2 **Protein sequence alignment**. Concatenated amino-acid sequence alignment of the 13 mitochondrial protein-coding genes (in Nexus format) used in the phylogenetic analyses. Click here for file

Additional file 3 **DNA sequence alignment**. Concatenated nucleotid sequence alignment of the 13 mitochondrial protein-coding genes (in Nexus format) used in the branch length analysis. Click here for file

Additional file 4 **DNA sequence alignment**. Concatenated rRNA sequence alignment (in Nexus format) used in the branch length analysis. Click here for file
